# The clinical and multimodal imaging features of IgG4-related dacryoadenitis with coexisting vitiligo: A case report

**DOI:** 10.1097/MD.0000000000047892

**Published:** 2026-03-06

**Authors:** Tian Tian, Liguo Feng

**Affiliations:** a Department of Ophthalmology, Sir Run Run Shaw Hospital, Zhejiang University School of Medicine, Hangzhou, Zhejiang, China.

**Keywords:** histopathology, IgG4-related dacryoadenitis, ultrasound

## Abstract

**Rationale:**

IgG4-related dacryoadenitis is an ocular manifestation of IgG4-related disease. Reports on its multimodal imaging features, particularly the manifestations on color Doppler ultrasound, are rare.

**Patient concerns:**

A 76-year-old woman experienced persistent edema of the bilateral upper eyelids for more than 2 years, with a medical history of vitiligo, allergic dermatitis, and hypertension. Physical examination revealed enlarged lacrimal glands with palpable, firm, and movable nodules, accompanied by swollen eyelids and swollen lymph nodes in front of the ears. Color Doppler ultrasound showed the lacrimal glands were markedly enlarged bilaterally, rich in blood supply. Orbital computed tomography highlighted diffuse enlargement of the lacrimal glands bilaterally, with slightly higher density. Serum IgG4 levels were elevated.

**Diagnoses:**

IgG4 related dacryoadenitis.

**Interventions:**

A biopsy of left lacrimal gland was performed and methylprednisolone 20 mg/day was prescribed and then tapered.

**Outcomes:**

Her symptoms improved significantly 1 week after treatment. Serum IgG4 levels gradually decreased. At the 6-month follow-up, the condition was stable.

**Lessons:**

IgG4-related dacryoadenitis should be considered in the differential diagnosis of unexplained lacrimal gland enlargement. The association between IgG4-related dacryoadenitis and concurrent vitiligo requires further investigation.

## 1. Introduction

IgG4-related dacryoadenitis is a common ocular manifestation of IgG4-related disease (IgG4-RD). Its typical clinical features include symmetrical enlargement of the unilateral or bilateral lacrimal glands, which are relatively hard in texture and mostly painless. Some patients may have involvement of other glands or organs (such as salivary gland enlargement, pancreatic involvement, etc).^[1]^ However, reports on its multimodal imaging features, particularly the manifestations on color Doppler ultrasound, are rare. This case demonstrates the color Doppler ultrasound findings of the disease, while also presenting clinical features, pathological results, medium-term follow-up outcomes, aiming to provide valuable insights for clinical practice. This study is a single-case report that only involves the retrospective analysis of routine clinical diagnosis and treatment data without additional interventions. In accordance with the ethical guidelines for single-case reports of the institutional review board of the Sir Run Run Shaw Hospital, Zhejiang University School of Medicine, ethical committee approval is not required. Written informed consent for publication of the case details and related materials was obtained from the patient.

## 2. Case presentation

A 76-year-old female presented to the nephrology department with persistent edema of the bilateral upper eyelids for more than 2 years, with a medical history of vitiligo, allergic dermatitis, and hypertension. No typical associated lesions were detected by electrocardiogram, echocardiogram, carotid ultrasound, abdominal ultrasound (liver, gallbladder, pancreas, spleen), urinary system ultrasound, or chest computed tomography (CT). She was diagnosed with edema of undetermined etiology and prescribed multiple medications, though her symptoms persisted.

**Figure 1.**
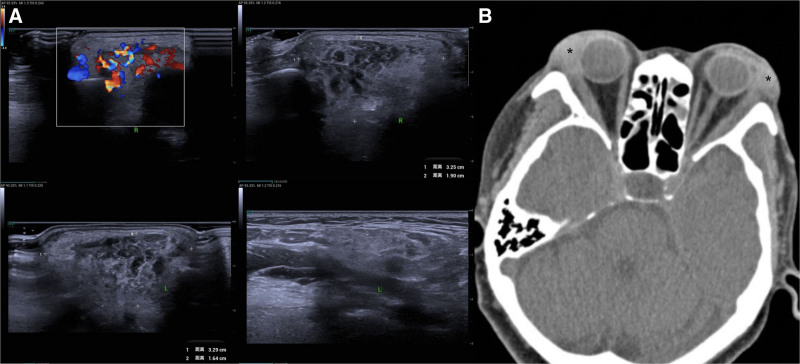
Imaging data. Color Doppler ultrasound reveals bilateral lacrimal glands that are plump, thickened, and diffusely enlarged, with the right measuring approximately 3.25 × 1.90 × 1.93 cm and the left approximately 3.29 × 1.64 × 2.12 cm. The glands exhibit relatively regular morphology with slightly ill-defined margins. Their internal echotexture is heterogeneous, showing hypoechoic to isoechoic signals interspersed with patchy hypoechoic areas and a reticular pattern. Marked hypervascularity is demonstrated, characterized by abundant blood perfusion (Panel A). Orbital computed tomography (CT) shows diffuse soft tissue edema in the superolateral orbital lacrimal gland region. The lacrimal gland is diffusely enlarged, with irregular morphology and ill-defined borders. Plain scan reveals uniform slight hyperdensity (mean Hounsfield unit ≈ 100, *marked), without definite hypodense lesions or calcific foci in the glandular parenchyma. Orbital wall bony structures are intact, with no destruction or proliferative changes (Panel B).

**Figure 2.**
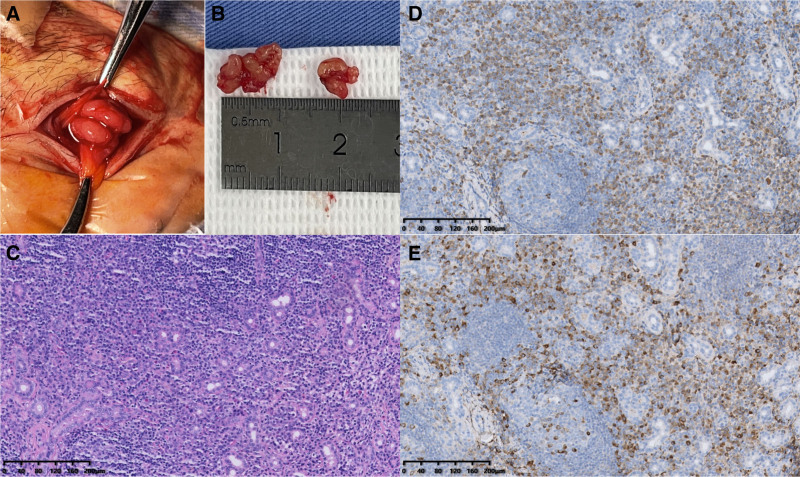
Intraoperative specimen and pathology images. Two lobulated, encapsulated nodules were identified within the enlarged lacrimal glands, which were firm and grayish-white, measuring 10 × 9 mm and 6 × 5 mm, respectively (Panels A and B). Hematoxylin–eosin staining revealed dense diffuse inflammatory cell infiltration, predominantly composed of lymphocytes and plasma cells (Panel C). IgG4 staining showed diffuse distribution of IgG4-positive plasma cells, with a count of >200/high-power field in hot spots (Panel D). IgG staining demonstrated diffuse strong positive expression in the abundant infiltrating plasma cells within the glandular parenchyma and stroma, with the ratio of IgG4-positive cells to total IgG-positive cells being ≥40% (Panel E).

The patient attended the ophthalmology department due to unimproved symptoms. Physical examination revealed localized skin depigmentation and non-pitting upper eyelid edema, with firm, poorly mobile, non-tender lacrimal gland enlargement. She underwent color Doppler ultrasound and orbital CT examinations. As shown by color Doppler ultrasound, the bilateral lacrimal glands are plump, thickened, and diffusely enlarged, with the right lacrimal gland measuring approximately 3.25 × 1.90 × 1.93 cm and the left lacrimal gland approximately 3.29 × 1.64 × 2.12 cm. The glands exhibit a relatively regular morphology with slightly ill-defined margins. The internal echotexture of the glands is heterogeneous, presenting hypoechoic to isoechoic signals with patchy hypoechoic areas and a reticular pattern. Color Doppler interrogation reveals marked hypervascularity with abundant perfusion (Fig. 1, Panel A). Orbital CT demonstrates diffuse soft tissue edema in the lacrimal gland region located at the superolateral aspect of the orbit. The lacrimal gland exhibits diffuse enlargement, characterized by an irregular morphology and ill-defined borders. On plain scan, the gland manifests as uniformly slightly hyperdense, with a mean Hounsfield unit value of approximately 100 (*marked). No definite hypodense lesions or calcific foci are detected within the glandular parenchyma. The bony structures of the orbital wall remain intact, without any signs of destruction or proliferative changes (Fig. 1, Panel B). Enhanced MRI was not performed due to non-removable metal dental implants. A lacrimal gland biopsy was subsequently performed. Intraoperatively, 2 lobulated, encapsulated masses (firm, grayish-white, 10 × 9 mm and 6 × 5 mm) were resected from the left lacrimal gland (Fig. 2, Panels A and B) The serum IgG4 level was 22.3 g/L (reference range, 0.03–2.01 g/L). Pathology revealed lymphoid hyperplasia with follicle formation, local fibrous hyperplasia, massive plasma cell infiltration, and increased IgG4-positive plasma cells in the mucous gland stroma. Immunohistochemical staining was positive for IgG4 (>200/high-power field in hot spots, IgG4+/IgG + ratio > 40%) (Fig. 2, Panels C–E). Histopathological findings revealed no monoclonal lymphocytic proliferation in the lesion tissue, with no significant upregulation in the expression of CD20, CD3, and Ki-67, and no lymphoma-specific immunophenotypic abnormalities. Combined with the imaging features of lacrimal gland-localized lesions with relatively regular morphology, the diagnosis of lacrimal gland lymphoma was excluded. No typical diffuse eosinophilic infiltration or massive neutrophil aggregation was observed in the pathological sections; in addition, serological tests showed no significant elevation of inflammatory markers such as erythrocyte sedimentation rate and C-reactive protein, thus ruling out idiopathic orbital inflammation. According to the 2023 revised diagnostic criteria, the patient was ultimately diagnosed with IgG4-related dacryoadenitis. The specific diagnostic crieria are showed in Table 1.^[1]^

**Table 1:** The 2023 revised diagnostic criteria for IgG4-related dacryoadenitis and sialadenitis.

1. Persistent (>3 mo) swelling of the lacrimal, parotid, and submandibular glands
a. Symmetrical, and 2 or more pairs b. One or more glands
2. Serologically high levels of IgG4 > 135 mg/dL (1.35 g/L)
3. Marked IgG4 + plasma cells infiltration (IgG4+/IgG + cells > 40% and IgG4 + plasma cells > 10/high-power field) into lacrimal and salivary gland tissues

IgG4-related dacryoadenitis and sialadenitis is defined as satisfying 1a + either 2 or 3, or 1b + 2 + 3.

Methylprednisolone (20 mg/day) was added to her regimen. Monthly follow-ups showed gradual symptom improvement, with corresponding improvements in ultrasound findings and IgG4 levels, and the dosage was tapered to 6 mg. At 3.5 months, the serum IgG4 level was 9.44 g/L. Color Doppler ultrasound showed that the bilateral lacrimal glands were significantly reduced in size. The right lacrimal gland measured approximately 1.91 × 1.11 × 1.19 cm, and the left lacrimal gland approximately 1.66 × 0.95 × 1.43 cm. Vascularization was also decreased compared with the previous examination. At the 6-month follow-up, the patient’s symptoms remained in sustained remission. Notably, the patient reported voluntarily discontinuing glucocorticoid therapy. Two days after cessation, eyelid swelling recurred: prompting her to promptly resume oral administration at the original dose independently, with symptoms resolving once more.

## 3. Discussion

The main feature of this patient is painless swelling of the bilateral eyelids without any systemic involvement. For such unexplained lacrimal gland enlargement, IgG4-RD should be considered in the differential diagnoses. Serum IgG4 testing and pathological biopsy are recommended when necessary to avoid misdiagnosis. Currently, detailed descriptions of lacrimal gland ultrasound findings in IgG4-related dacryoadenitis are rare. Ultrasound is low-cost, convenient, high-resolution, noninvasive, and radiation-free, with real-time dynamic imaging capabilities. It clearly visualizes the layered structure, lesion extent, and adjacent tissue relationships of the superficial orbital soft tissues, proving highly valuable for preliminary screening and dynamic follow-up of orbital diseases. The ultrasonic findings of IgG4-related dacryoadenitis reported in this case were characterized by relatively regular glandular morphology with ill-defined borders, heterogeneous internal echoes presenting as hypoechoic to isoechoic patterns, as well as abundant blood flow signals. This condition can be preliminarily differentiated from other orbital diseases, such as orbital lymphoma and idiopathic orbital inflammation. Orbital lymphoma usually involves regions beyond the lacrimal gland, mostly manifesting as hypoechoic masses with irregular shapes, heterogeneous internal echoes accompanied by cord-like hyperechoic foci and abundant blood flow signals.^[2]^ Idiopathic orbital inflammation presents nonspecific ultrasonic manifestations, mostly with ill-defined lesions and relatively homogeneous internal echoes; the intensity and distribution of blood flow signals vary considerably with different inflammatory stages.^[3]^ The references regarding the differential ultrasonic manifestations between IgG4-related dacryoadenitis and Sjögren syndrome-related dacryoadenitis remain scarce. Most reported imaging manifestations of Sjögren syndrome-related dacryoadenitis are based on CT or MRI, and further ultrasonic studies are warranted.^[4,5]^ Our case clearly demonstrates the ultrasound characteristics of the lacrimal gland in this disease, which may provide references for the imaging diagnosis of IgG4-related dacryoadenitis in the future. It should be emphasized that ultrasound serves as a tool for preliminary localization and auxiliary diagnosis of orbital lesions. The accuracy and reliability of diagnosis need to be improved by combining it with other examinations, such as serological examination, CT, MRI, and pathological biopsy.

Interestingly, our patient is complicated with vitiligo. Previously, only 1 case of IgG4-related dacryoadenitis complicated with vitiligo has been reported.^[6]^ Whether there is an association between the pathogenic mechanisms of IgG4-RD and vitiligo remains unknown currently. IgG4-RD comprises a group of autoimmune disorders characterized by IgG4+ plasma cell infiltration, tissue fibrosis, and multi-organ involvement. Polarized CD4+T cells drive this process by activating innate immune cells to promote fibrosis, alongside the production of IgG4-secreting plasma cells: ultimately leading to immune cell infiltration and tissue damage.^[7]^ Vitiligo arises from CD8 + cytotoxic T cells targeting and eliminating melanocytes, combined with regulatory T cell dysfunction and imbalances in CD4 + T cell subsets. This results in the selective loss of melanocytes, impaired capacity to suppress autoreactive immunity, and exaggerated inflammatory damage.^[8]^ Both IgG4-RD and vitiligo involve the aberrant activation of CD4 + T cells. Furthermore, IgG4-RD and vitiligo have each been reported to be associated with HLA-DRB1 gene polymorphism.^[9,10]^ The comorbidity of IgG4-RD and vitiligo holds certain clinical significance, suggesting that the 2 conditions may share common genetic susceptibility and underlying immune dysregulation. These commonalities may provide insights into exploring disease pathogenesis: overlapping immune pathways are hypothesized to be one of the causes for the co-occurrence of these 2 seemingly independent autoimmune disorders. Further in-depth, large-sample, multi-center studies are warranted to investigate whether there exist shared pathogenic correlations and genetic susceptibility loci between them, thereby laying a theoretical foundation for the development of targeted therapies.

The international consensus guidance classifies glucocorticoids (GCs) as 1st-line therapy for IgG4-RD remission induction, with core principles including pretreatment biopsy to rule out malignancy and maintenance therapy for high-risk patients.^[11]^ GCs demonstrate significant efficacy in most patients, improving clinical manifestations, reducing organ enlargement, and lowering serum IgG4 levels.^[12]^ However, the recurrence rate during GCs tapering or maintenance therapy ranges from 15% to 60%.^[13]^ Disease-modifying antirheumatic drugs (DMARDs) act as steroid-sparing agents for mild-to-moderate disease but cannot induce remission alone.^[14]^ Rituximab, a CD20-targeted monoclonal antibody depleting B lymphocytes, is recommended for GC-refractory or intolerant patients,^[14–17]^ with recent evidence supporting its efficacy, safety, and role in reducing recurrence when used as maintenance therapy post-remission.^[18]^

Consistent with current guidelines, the patient received GCs as 1st-line therapy and achieved a favorable response. During the low-dose GCs maintenance phase, the patient’s condition remains stable without steroid-related adverse effects. However, the patient reported having voluntarily discontinued glucocorticoid therapy. Two days after the withdrawal, eyelid swelling recurred, and the patient promptly resumed oral administration at the original dose on her own, with symptoms resolving again. This clinical course indicates that the patient exhibits the clinical feature of steroid dependence. Abrupt discontinuation of GCs would increase the risk of disease recurrence, whereas long-term maintenance of glucocorticoid treatment will lead to higher cumulative steroid exposure, thereby elevating the incidence of adverse reactions such as osteoporosis, glycemic disorders, and gastrointestinal ulcers. During subsequent follow-ups, if the patient experiences poor disease control, increased frequency of relapses, difficulty in steroid tapering, or steroid-related adverse events, we will initiate rituximab therapy to strike a balance between disease control and minimizing steroid-associated risks. Owing to the single-case nature of this report and the short follow-up duration, the long-term prognosis, risk of disease recurrence, and potential progression to systemic IgG4-RD of this patient remain unclear. These limitations also indicate that future large-sample, long-term prospective studies are warranted to clarify the long-term outcomes of such cases.

## Author contributions

**Supervision:** Liguo Feng.

**Writing – original draft:** Tian Tian.

**Writing – review & editing:** Liguo Feng
